# Characterization of the mating systems *of Anopheles gambiae* in malaria control perspective

**DOI:** 10.1186/1475-2875-11-S1-O42

**Published:** 2012-10-15

**Authors:** Abdoulave Diabaté

**Affiliations:** 1 Institut de Recherche en Sciences de la Santé/Centre Muraz, Bobo-Diolasso, Burkina Faso

## 

The current works is focussed on ways in which the manipulation of mosquito mating behaviour has the potential to contribute to integrated programme for the control of malaria vectors. One line of investigation explores means of reducing vector populations by significantly reducing mating with lure-and-kill and mating disruption measures. The second line is looking at variation in mating success between and within swarms of *Anopheles gambiae* and its underlying factors with an ultimate goal of designing mosquito rearing scheme that produce competitive mating males.

A complete map of swarm distribution in Vallée du Kou was constructed and swarms were physically described. Overall swarms were tightly linked to specific man-made markers within the village and a significant difference in swarm numbers and size was observed between households. The pattern distribution of swarms across space was clustered and hotspots are clearly seen where most of the swarms aggregate. A multivariate analysis allowed identifying a subset of environmental parameters that best correlate to swarm structures and that includes, the number of swarm markers/surface unit, the exposition of the makers to sunlight, the contrast pattern and the openness of the marker to air circulation. Exploration of the energetic budget in relation to swarming and mating showed that sugars and glycogen are the main energetic sources that fuel males mating activities. The distribution of wing size of mated males was focused around a central value suggesting that intermediate size of males is advantageous in *An. gambiae* mating system.

A better knowledge of key parameters that account for male mating success will be of significance to control strategies based on the release of genetically modified or sterilised males. Similarly, understanding the ecological parameters that are correlated with the presence or absence of swarms would be valuable for the implementation of mating disruption strategies.

